# Patellofemoral arthroplasty in isolated Iwano grade IV patellofemoral osteoarthritis: 86% 10‐year survival

**DOI:** 10.1002/jeo2.70515

**Published:** 2025-11-28

**Authors:** Yu‐Hung Tian, Kuan‐Lin Chen, Pai‐Han Wang, Po‐Kuei Wu, Cheng‐Fong Chen, Wei‐Ming Chen

**Affiliations:** ^1^ Department of Orthopedics & Traumatology, Division of Joint Reconstruction Taipei Veterans General Hospital Taipei Taiwan; ^2^ Therapeutical and Research Center of Musculoskeletal Tumor Taipei Veterans General Hospital Taipei Taiwan; ^3^ Department of Orthopedic Surgery, School of Medicine National Yang Ming Chiao Tung University Taipei Taiwan; ^4^ Department of Orthopaedic Surgery Stanford University School of Medicine Redwood City California USA

**Keywords:** patellofemoral arthroplasty, patellofemoral osteoarthritis, total knee arthroplasty

## Abstract

**Purpose:**

Isolated patellofemoral osteoarthritis (PFOA) is a degenerative condition, typically presenting as anterior knee pain. For patients who are refractory to conservative treatment, patellofemoral arthroplasty (PFA) is a viable surgical option. Appropriate patient selection is fundamental to achieving favourable long‐term outcomes. Large multicenter and registry studies have reported outcomes of PFA (under 0.45%–2% utilization). Howevever, because of the heterogenicity of indications and surgeons, those findings may not be transferable to individual surgeon practices. This study aimed to evaluate the utilization and long‐term survival of PFA within a high‐volume, single‐surgeon arthroplasty practice.

**Methods:**

This retrospective study included consecutive cases of Patellofemoral arthroplasties performed between 2012 and 2014. Patients with prior ipsilateral knee surgery, patellar instability, or less than 10 years of follow‐up were excluded. The number of primary knee arthroplasties performed within the same period was collected. Radiographic assessments included Kellgren–Lawrence grading, Iwano grading, Insall‐Salvati ratio, Dejour classification, medial proximal tibial angle, joint line convergence angle, sulcus angle and anatomical lateral distal femoral angle. Implant survival rates were evaluated over a 10‐year follow‐up period.

**Results:**

A total of 37 patients (50 patellofemoral arthroplasties) who underwent PFA for isolated PFOA were included, alongside 2556 primary knee arthroplasties. PFAs accounted for 2.0% of the primary knee arthroplasties performed during the study period. All patients were classified as Iwano grade IV preoperatively. The 10‐year survival rate was 86.0%. All seven PFA failures were due to progression of tibiofemoral osteoarthritis and were converted to total knee arthroplasty.

**Conclusions:**

PFA demonstrated a 10‐year implant survival rate of 86.0% when performed under strict selection criteria in a high‐volume arthroplasty centre. Achieving optimal outcomes requires careful patient selection, surgical proficiency and alignment with patient expectations. Further prospective studies are warranted to better evaluate long‐term functional outcomes after PFA.

**Level of Evidence:**

Level III, retrospective cohort study.

**Abbreviations**
aLDFA: anatomical lateral distal femoral angle
AP: anteroposterior
BMI: body mass index
IRB: institutional review board
JLCA: joint line convergence angle
K‐L: Kellgren–Lawrence
MPTA: medial proximal tibial angle
PACU: postanaesthesia care unit
PFA: patellofemoral arthroplasty
PFOA: patellofemoral osteoarthritis
TFOA: tibiofemoral osteoarthritis
TKA: total knee arthroplasty
VAS: visual analogue scale
WOMAC: Western Ontario and McMaster Universities Osteoarthritis Index

## INTRODUCTION

Isolated symptomatic patellofemoral osteoarthritis (PFOA) typically presents with anterior knee pain that worsens with activities such as squatting and occurs without significant tibiofemoral joint space narrowing, affecting about 2% of men and 8% of women over 55 years old [41]. PFOA is mostly treated conservatively. For advanced PFOA patients refractory to conservative treatment, patellofemoral arthroplasty (PFA) has been utilised as a surgical option used to treat specific conditions within the PF joint, including isolated PFOA, posttraumatic arthritis, trochlear dysplasia and recurrent patellar dislocation [2, 6]. For patients with isolated PFOA, PFA offers a targeted, less invasive alternative to total knee arthroplasty (TKA) [5, 9, 23, 37]. Some studies reported higher forgotten knee score following PFA compared to TKA [35, 36]. However, conversion to TKA remains an undesirable outcome. The 10‐year survival rate has been reported to be around 75% in previous studies [21, 25]. The primary reason for subsequent conversion to TKA is the progression of tibiofemoral osteoarthritis (TFOA) [19, 29]. Multiple surgeon‐related factors have been reported to be associated with long‐term survival of arthroplasty, including strict patient selection and high surgical volume [4, 16, 17, 22, 34, 42]. Since the indication for PFA remains narrow, there are limited data on its utilization, specifically, the percentage of PFAs among all primary knee arthroplasties performed using consistent inclusion criteria that only include Iwano grade IV patients, and the corresponding clinical outcomes [14].

This study, therefore, aimed to report the percentage of PFA utilization among all primary arthroplasy service in a high‐volume single‐surgeon practice, where the indication for PFA was limited to Iwano grade IV osteoarthritis without patellar instability, and to evaluate its correlated outcomes. It was hypothesised that PFA, when performed under strict selection criteria in a high‐volume practice, would demonstrate satisfactory long‐term implant survival and functional improvement comparable to previously reported outcomes.

## MATERIALS AND METHODS

### Study population data collection

**Figure 1:**
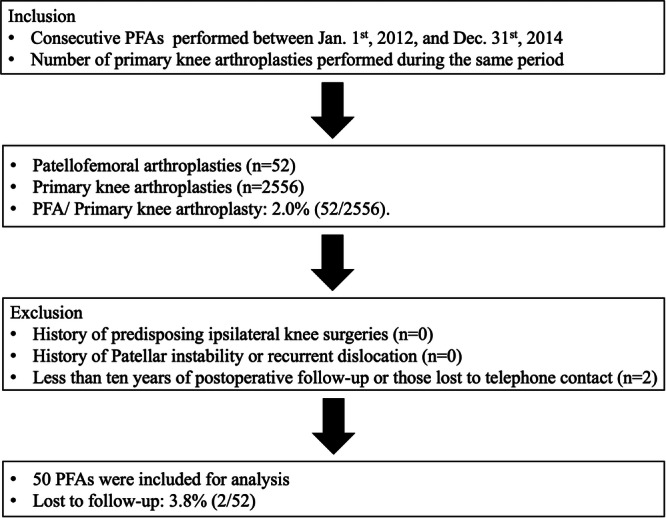
Patient selection flowchart. Flowchart demonstrating the patient selection process. Inclusion and exclusion criteria were applied sequentially to identify eligible patients for the final study cohort. PFA, Patellofemoral arthroplasty. TKA, total knee arthroplasty.

This retrospective cohort study was approved by the Institutional Review Board (IRB) of Taipei Veterans General Hospital (IRB No.: 2025‐01‐030AC) and was conducted in accordance with the STROBE guidelines and the principles of the Declaration of Helsinki. It involved a review of consecutive patients who underwent PFA performed by a single surgeon at a tertiary medical centre between 1 January 2012, and 31 December 2014. Informed consent was obtained from all individual participants included in the study. A single‐surgeon study design was chosen to ensure consistency in surgical technique, standardisation in clinical decision‐making, and uniformity in patient selection criteria. Exclusion criteria included: (1) patients with a history of predisposing ipsilateral knee surgeries, infections, fractures, rheumatoid arthritis, or lower limb tumours prior to PFA; (2) patients with patellar instability or recurrent dislocation and (3) patients with less than 10 years of postoperative follow‐up or those lost to telephone contact. Comprehensive demographic data were systematically collected and organised. The total volume of primary TKA was calculated, and the proportion of PFA cases among all TKA procedures during the same inclusion period was analysed. The general indications for PFA in this practice included isolated advanced patellofemoral arthritis (Iwano grade IV) with positive gliding test that has refractory to conservative treatment including lifestyle modification, physical therapy, muscle strengthening exercise, intra‐articular hyaluronic acid injections and pain medications for over 6 months. A comprehensive summary of the patient selection process is presented in Figure 1. Patients were excluded from PFA if they had systemic inflammatory arthropathy, TFOA greater than Kellgren‐Lawrence (K–L) Grade II, uncorrected patellofemoral instability or malalignment, tibiofemoral mechanical malalignment (valgus >8° or varus >5°), active infection, or significant loss of knee range of motion [14]. All PFAs were reviewed and approved by committee for appropriate indications preoperatively. Additionally, the preoperative visual analogue scale (VAS) and Self‐administered Western Ontario and McMaster Universities (WOMAC) index were collected [3, 12].

### Radiographic evaluations

Radiographic data were obtained from the hospital's imaging system. Routine preoperative imaging included standing anteroposterior (AP) and lateral views of the knee, as well as 45‐degree Merchant views, which were used for both preoperative and postoperative assessments. Preoperative standing knee AP views were analysed to determine the K–L grade, medial proximal tibial angle (MPTA), anatomical lateral distal femoral angle (aLDFA) and joint line convergence angle (JLCA) [15, 26]. The standing knee lateral view was used to calculate the Insall‐Salvati ratio for assessing patellar position [13]. Trochlear dysplasia was evaluated using the Dejour classification system based on both the standing knee lateral and 45‐degree Merchant views [10]. PFOA was classified using the Iwano system on a 45‐degree Merchant view [14]. Additionally, the sulcus angle, defined as the angle formed by lines connecting the highest points of the femoral condyles to the deepest point of the trochlear groove, was measured and recorded as part of the preoperative evaluation. All radiographic measurements were performed independently by an orthopaedic specialist. Intraobserver reliability was assessed in 20 randomly selected cases, and the intraclass correlation coefficient (ICC) demonstrated excellent repeatability (ICC = 0.90).

### Surgical technique and postoperative care

Patients underwent either unilateral or bilateral PFA (Gender Solutions™ Patello‐Femoral Joint System, a second‐generation onlay‐type prosthesis) under spinal or general anaesthesia. A midvastus approach was utilised to access the knee joint without damaging the anterior horn of the meniscus, and electrocautery was used to remove the infra‐patellar fat pad to enhance exposure. Femoral component was prepared by aligning anatomical landmarks, performing an anterior femoral cut using an intramedullary guide, and milling the trochlear groove with a sizing‐specific guide. Peg and tail holes were drilled for implant fixation. The patella was meticulously resurfaced using a measured resection technique to balance between maintaining bony strength and overstuffing. First, the patellar thickness was measured. Second, the target thickness was calculated by subtracting the thickness of the patellar component from the measured value. If the patellar cartilage was severely worn, 2 mm was added back to the target thickness. If the calculated target thickness was less than 12 mm, a minimum of 12 mm was maintained to ensure sufficient bony strength. Finally, the patella was resurfaced to the target thickness. After trialing and confirming proper alignment and patellofemoral tracking, the components were cemented in place. After adequate irrigation, the wound was closed by layers. Postoperatively, the position of the implant was confirmed with portable X‐ray in the postanaesthesia care unit (PACU) for documentation. Patients were mobilised on the same day, with full weight‐bearing and range of motion. No activity restriction was applied postoperatively. At discharge, patients were provided with a phone number that could be reached during office hours for any surgery‐related concerns. This line is dedicated exclusively to postoperative care within the surgeon's practice for better accessibility. Patients were scheduled to return to the clinic 2 weeks postop for wound check and 1 month, 3 months and 1 year postoperatively for radiographical and functional evaluation.

### Data analyses

Statistical analysis was conducted using SPSS version 25.0. Descriptive statistics, including median, mean and standard deviation, were used to summarise the data. The Mann–Whitney *U* test was employed to compare continuous variables between groups, and Fisher's exact test was utilised for comparisons of categorical variables. A *p*‐value of <0.05 was considered statistically significant. Kaplan–Meier survival analysis was performed to estimate survival rates.

## RESULTS

### Patient demographics

Between 1 January 2012, and 31 December 2014, a total of 39 patients underwent 52 PFA procedures. Of the 52 PFAs, 50 met the inclusion criterion of a minimum 10‐year follow‐up, yielding a loss to follow‐up rate of 3.8%. During the same period, the surgeon performed 2556 primary knee arthroplasties, of which PFA accounted for 2.0% of all cases.

### Survival of PFA, complications and functional evaluations

**Figure 2:**
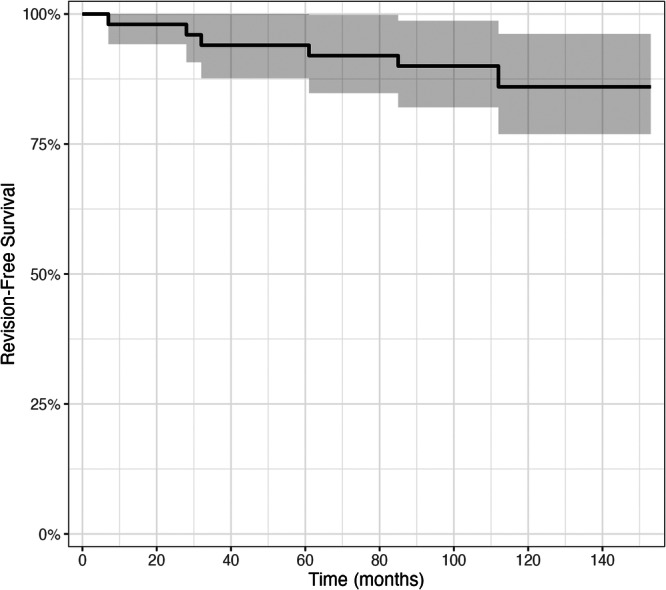
Patellofemoral arthroplasty (PFA) Kaplan–Meier survival curves across 10 years. The survival rate using Kaplan–Meier analysis shows that for 50 PFA surgeries, the 5‐year revision‐free survival was 94.0%. After 10 years, 43 knees were still preserved, with a revision‐free survival of 86.0%.

In this series, the 5‐year revision free survival rate for PFA was 94.0%, and the 10‐year revision free survival rate was 86.0%. All seven cases of conversion to TKA were attributable to the progression of TFOA, with four knees (57.1%) predominantly involving the lateral compartment and three knees (42.9%) predominantly involving the medial compartment. The Kaplan–Meier survival curve for PFA is shown in Figure 2. No complications, including periprosthetic infection, wound dehiscence, urinary retention, or thromboembolic events, was recorded. All patients showed substantial improvement in functional and pain scores. At the 1‐month postoperative follow‐up, no patient required additional new pain medication prescriptions.

### Conversion group versus nonconversion group

There were no significant differences in the preoperative demographic data between the conversion group and nonconversion group, as detailed in Table 1.

**Table 1:** Demographic data between the nonconversion group and the conversion group.

	Nonconversion group (*N* = 31)	Conversion group (*N* = 6)	*p*‐Value
Patient number (*N* = 37)	83.7% (31)	(6) 16.3%	
Age	72.2 ± 9.4	72.3 ± 5.1	b0.90
Female	90.3% (28)	100.0% (6)	a0.98
Bilateral PFA	38.7% (12)	16.7% (1)	a0.48
BMI	25.9 ± 4.3	27.1 ± 7.3	b0.56
Diabetic mellitus	22.6% (7)	33.3% (2)	a0.64
Preoperative VAS	6.5 ± 2.1	6.6 ± 1.9	b0.94
Preoperative WOMAC index			
Pain	17.8 ± 2.4	17.0 ± 1.9	b0.67
Stiffness	5.4 ± 1.1	4.9 ± 1.1	b0.12
Physical function	51.5 ± 4.2	53.7 ± 4.2	b0.25

Abbreviations: BMI, body mass index; PFA, patellofemoral arthroplasty; VAS, visual analogue scale; WOMAC index, Western Ontario and McMaster Universities index.

^a^ Fisher's exact test.

^b^ Mann–Whitney *U* test.

The nonconversion group, consisting of 31 patients, 43 PFAs, had an average age of 72.2 ± 9.4 years. 90.3% (28/31) of patients were female. Among this group, 38.7% (12/31) underwent bilateral PFA. Preoperatively, the patients had a mean VAS score of 6.5, with the WOMAC index as follows: pain subscale 17.8 points, stiffness 5.4 points and physical function 51.5 points. According to the preoperative K–L grading system, 90.7% of patients' knees were classified as grade I, while 9.3% were classified as grade II. Preoperative radiographic measurements revealed a mean MPTA of 87.3 ± 1.9 degrees, a mean aLDFA of 79.6 ± 2.6 degrees, a mean JLCA of 2.4 ± 1.5 degrees, and a mean sulcus angle in axial view of 134.8 ± 8.4. Regarding patellar positioning, lateral weight‐bearing knee X‐rays showed an Insall‐Salvati ratio of 1.20 ± 0.17. The Dejour classification of trochlear dysplasia indicated that 40 patients’ knee (93.0%) were classified as grade I, and 3 patients’ knee (7.0%) as grade II (Table 2).

**Table 2:** Radiographic parameters comparison between the nonconversion group and the conversion group.

	Nonconversion group (*N* = 43)	Conversion group (*N* = 7)	*p*‐Value
Preoperative TFOA K‐L Grade I/Grade II	93.0% (40)/7.0% (3)	85.7% (6)/14.3% (1)	a0.93
PF Iwano Grade IV	100.0% (43)	100.0% (7)	a1.00
MPTA	87.3 ± 2.0	87.3 ± 2.7	b0.53
aLDFA	79.6 ± 2.6	76.9 ± 3.1	b0.02*
JLCA	2.4 ± 1.5	2.2 ± 1.5	b0.78
Insall‐Salvati Ratio	1.20 ± 0.17	1.04 ± 0.17	b0.03*
Sulcus Angle (45 degrees)	134.8 ± 8.4	131.8 ± 4.2	b0.42
Dejour Grade I	95.3% (41)	71.4% (5)	a0.26

Abbreviations: aLDFA, anatomical lateral distal femoral angle; JLCA, joint line convergence angle; K–L grade, Kellgren–Lawrence (K–L) grade; MPTA, medial proximal tibial angle; PF Iwano grade, patellofemoral arthritis Iwano grade; TFOA, tibiofemoral osteoarthritis.

^a^ Fisher's exact test.

^b^ Mann–Whitney *U*‐Test.

* The difference was statistically significant (*p* < 0.05).

The conversion group, consisting of six patients, seven PFA procedures, had an average age of 72.3 ± 5.08 years, all patients were female. Among the six patients, one patient underwent bilateral PFA. The reason for implant removal in all patients was conversion to TKA due to progression of TFOA. Preoperatively, these patients had a mean VAS score of 6.6, a WOMAC pain subscale of 17.0 points, stiffness of 4.9 points, and a physical function score of 53.7 points. The preoperative K–L grade revealed that six cases (86.8%) had grade I osteoarthritis, while one case (14.2%) had grade II. The preoperative radiographic measurements for this group included a mean MPTA of 87.2 ± 2.3 degrees, a mean aLDFA of 76.9 ± 3.2 degrees, a mean JLCA of 2.2 ± 1.5 degrees and a mean sulcus angle in axial view of 131.8 ± 4.2 degrees. For patellar positioning, lateral weight‐bearing knee X‐rays showed an Insall‐Salvati ratio of 1.04 ± 0.17, and according to the Dejour classification of trochlear dysplasia, five cases (71.4%) were classified as grade I, and two cases (28.6%) as grade II (Table 2).

**Figure 3:**
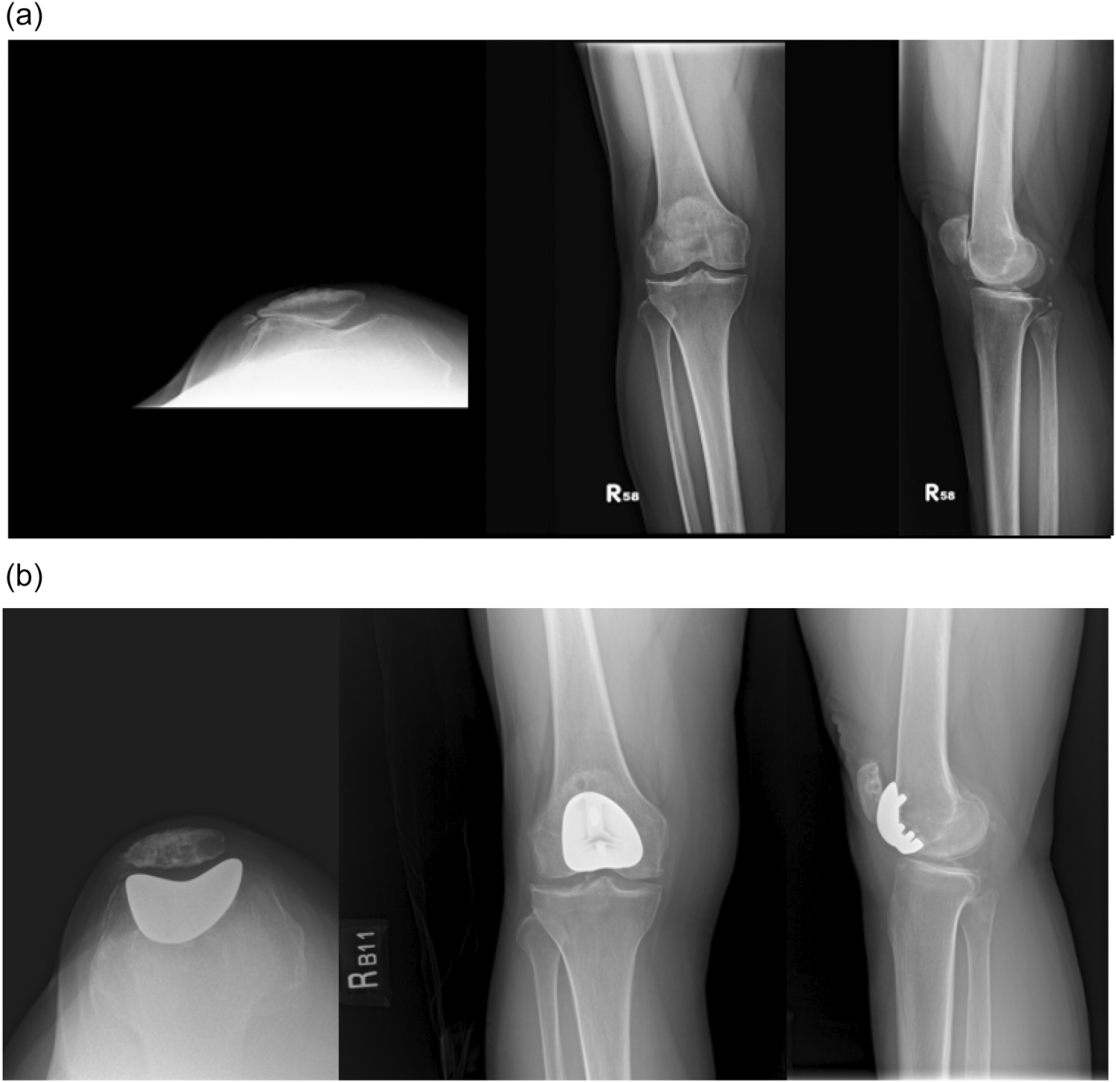
(a) A 57‐year‐old female underwent right patellofemoral arthroplasty (PFA) in 2014 due to patellofemoral arthritis in the right knee (Iwano classification grade IV). Preoperative imaging included standing knee X‐ray and Merchant view at 45 degrees. (b) The X‐ray findings of the patient 10 years postoperatively are presented. The knee joint space narrowing was acceptable despite noted progression of tibiofemoral osteoarthritis.

**Figure 4:**
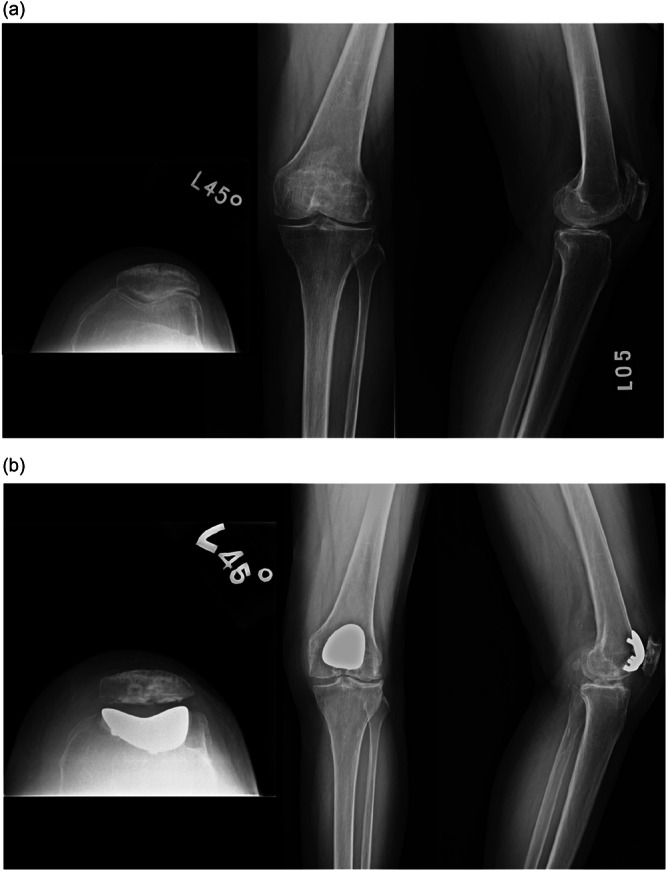
(a) A 68‐year‐old female underwent patellofemoral arthroplasty (PFA) in 2014. Preoperative plain radiographs showed grade IV patellofemoral arthritis, a small anatomical lateral distal femoral angle (aLDFA), and a lower Insall–Salvati ratio. (b) Three years later, the progression of tibiofemoral osteoarthritis led the patient to undergo total knee arthroplasty (TKA).

Figures 3 and 4 illustrate representative cases from the nonconversion and conversion groups, respectively. Radiographic analysis showed that the conversion group had significantly smaller aLDFA and lower Insall‐Salvati ratio, with *p*‐values of 0.02 and 0.03, respectively (Table 2). Other than these, there were no significant differences in demographic or preoperative variables, including age, BMI, gender, TFOA K–L grade, PF Iwano grade, MPTA, sulcus angle or Dejour grade.

## DISCUSSION

The main findings of this study are a 94.0% PFA survival rate at 5 years and 86.0% at 10 years in a highly selective cohort of Iwano grade IV patients, representing 2.0 % of the 2556 primary TKAs performed during the same period, with no perioperative complications observed. All cases demonstrated significant pain relief and functional improvement following the surgery. Despite the strict indication in a high‐volume practice, 10‐year survival was not much different from current literature. List et al. reported PFA survival rates of 91.7% at 5 years and 83.3% at 10 years, while Pagano et al. reported a 10‐year survival rate of 79% [25, 37]. In this study, 14% of PFA received conversion to TKA within 10 years. The leading reason for conversion was TFOA progression (100%). This is consistent with current findings [8, 18, 19]. In a large multination registry study, the most prevalent reason for revision PFA was TFOA progression consisting 42% (434 of 1034) of all revisions. Other reasons reported were persistent pain (17%, 176 of 1034), implant loosening or lysis (14%, 146 of 1034), malalignment and mal‐tracking (6%, 62 of 1034), patella implant breakage or wear (5%, 48 of 1034), instability or dislocation (4%, 41 of 1034), infection (3%, 31 of 1034) and fracture (1%, 10 of 1034) [19]. Those other conditions were not encountered in this series.

The 10‐year survival of this series is lower than that of TKA, as reported in historical results and published literature [8, 43]. Interestingly, the average age (72.2 years old) of the cohort was older than many published studies [11, 25]. Jonbergen et al. reported 10 year‐survival of 84% among a cohort averaged 52 years old [38]. The older patient population in this study may be attributed to the reimbursement criteria of national health insurance, which specifically requires patients to have a K–L grade ≥3 in at least two knee compartments to qualify for TKA. Isolated PFOA in patients over 70 years old would not be eligible for reimbursement for TKA. Therefore, PFA became their surgical alternative in older isolated PFOA patients. The survival in this study, despite being older in age, was comparable to that in the published literature.

In this study, PFA constitutes of 2.0% of all primary total knee arthroplasties. Larger registry or multicenter studies have reported PFA accounts for approximately 0.45%–2% of primary knee arthroplasty [19, 24]. However, the results of those studies may not be transferable to individual practices. PFAs may be concentrated among specific surgeons within a group of arthroplasty specialists. This study is the first to report the proportion PFA among primary TKAs in a high‐volume single surgeon practice and its correlating long‐term survival. Pappas et al. reported that a significant portion of total knee arthroplasties in the US are done by surgeons performing less than 50 TKAs per year and outcome of arthroplasty was associated with annual volume [27, 34]. Based on the 2.0% proportion from this study, this translates to fewer than one PFA case per year, a volume that may be insufficient for maintaining procedural proficiency. On the other hand, it could indicate that PFAs should be done in high‐volume services and better to be concentrated on a designated surgeon among the team for fluency. Performing a balanced, well‐symmetric patellar resurfacing was considered technically demanding, and as of now, it cannot be assisted by robotics. As the landscape trending towards selective patella resurfacing or even nonresurfacing, this technique may be further diminished among arthroplasty surgeons [1].

The revision rate for PFA is higher compared to TKA in similar patient populations [7, 33]. Therefore, there is concern that TKA may be more appropriate than PFA for older patients with isolated PFOA. However, recent studies have reported a more favourable safety profile and fewer perioperative complications associated with PFA [33, 40]. Studies have reported reliable improvement in functional outcomes after PFAs. Pogorzelski et al. reported 94% of survived PFAs could return to the same or higher level of sports [30]. However, the functional outcome may not be superior to TKA [32]. Lin et al. reported PFA to have higher forgotten knee score than TKA [20]. In summary, compared to TKA, PFA has moderately inferior long‐term implant survival, similar functional recovery but safer surgical profile compared to TKA. For patients requiring conversion to TKA from PFA, studies reported the outcomes were similar to primary TKA [28, 39]. Therefore, the decision to proceed with TKA or PFA for patients with isolated PFOA, particularly in older individuals, requires a thorough understanding of the patient's values to support effective shared decision‐making.

This study is limited by its retrospective design and relatively small sample size. The findings may also be subject to selection bias, as all patients were treated at a single high‐volume centre, which may limit the generalisability of the results to lower‐volume practices. However, although prior studies have suggested that the design of the PFA may influence the progression of TFOA, the conclusions of this study cannot be generalised to all types of implant designs [31]. Additionally, since the study included only Iwano grade IV patients and excluded PFAs performed for patellar instability, the data reflect only a subset of the broader indications for PFA.

## CONCLUSIONS

In conclusion, PFA, when performed under strict selection criteria in a high‐volume arthroplasty service, achieved a 10‐year implant survival rate of 86.0%. For patients with isolated patellofemoral osteoarthritis, in addition to adhering to strict indications, surgeons should consider their own technical proficiency and the patient's individual value system to achieve satisfying outcomes. For future functional assessment after PFA, more prospective studies are needed to provide in‐depth investigation.

## AUTHOR CONTRIBUTIONS

**Yu‐Hung Tian**: Writing—original draft; investigation. **Kuan‐Lin Chen**: Writing—review & editing; conceptualisation, methodology, supervision, project administration, validation. **Pai‐Han Wang**: Writing—review & editing. **Po‐Kuei Wu**: Data curation; formal analysis. **Cheng‐Fong Chen**: Data Curation; formal analysis. **Wei‐Ming Chen**: Conceptualisation; supervision, resources.

## CONFLICT OF INTEREST STATEMENT

The authors declare no conflict of interest.

## ETHICS STATEMENT

The research protocol was approved in advance by the appropriate ethical committee. Trial registration number and agency: TPEVGH IRB No.: 2025‐01‐030AC. All procedures performed in this study involving human participants were conducted in accordance with the ethical standards of the institutional and/or national research committee and with the 1964 Helsinki Declaration and its later amendments or comparable ethical standards. Informed consent was obtained from all individual participants included in the study. Patients signed informed consent regarding publishing their data and photographs.

## Data Availability

The datasets used and/or analysed during the current study are available from the corresponding author on reasonable request.
